# Degradation-Robust Hue Prior Network for Low-Light Rainy Image Restoration

**DOI:** 10.3390/s26154852

**Published:** 2026-08-01

**Authors:** Pujing Hu, Yixiao Liu, Xiaodong Luo, Chao Ren

**Affiliations:** 1 College of Electronics and Information Engineering, Sichuan University, Chengdu 610065, China; 2023141450138@stu.scu.edu.cn; 2 Sichuan Institute of Aerospace Electronic Equipment, China Aerospace Science and Technology Corporation, Chengdu 610199, China; lyx199177@163.com; 3 School of Artificial Intelligence, Sichuan Tourism University, Chengdu 610100, China; luoxd@sctu.edu.cn

**Keywords:** low-light rainy image restoration, hue prior, channel shuffle, adaptive feature modulation

## Abstract

Restoring images captured in low-light rainy scenes is challenging because brightness degradation and rain corruption are strongly coupled. Enhancing visibility may amplify hidden rain streaks and noise, whereas aggressive deraining can suppress already weak scene structures. Existing cascaded pipelines and general restoration models often struggle to handle this interaction effectively. In this paper, we present the Degradation-Robust Hue Prior Network (DHP-Net), a single-stage framework for low-light rainy image restoration that combines degradation-robust hue prior guidance with perturbation-aware feature modulation. Specifically, DHP-Net extracts multi-scale hue priors to provide stable structural and color cues under coupled degradations, and it injects them into a hierarchical Transformer restoration backbone. To further improve interaction among entangled feature responses, we introduce a Channel-adaptive Attention Perturbation Module that reorganizes intermediate representations before cross-channel aggregation. In this way, the proposed model jointly promotes visibility enhancement, rain removal, and structure preservation within a unified architecture. Extensive experiments on the Low-Light Rain (LLR) benchmark show that DHP-Net achieves 33.14 dB Peak Signal-to-Noise Ratio (PSNR) and 0.9252 Structural Similarity Index Measure (SSIM) on synthetic data and also delivers superior perceptual quality on real-world low-light rainy images, consistently outperforming existing state-of-the-art restoration models.

## 1. Introduction

Image restoration in adverse nighttime weather is difficult because the loss of visibility is caused by multiple interacting factors rather than a single corruption. In low-light rainy scenes, under-exposure suppresses contrast and weakens scene structures, while rain streaks further distort local textures and complicate brightness recovery. This coupled setting is therefore substantially different from conventional deraining [1,2,3,4,5,6], low-light enhancement [7,8,9,10,11,12], and generic restoration [13,14,15,16,17,18,19], which are typically optimized for isolated degradations.

Dedicated low-light rainy restoration has only recently begun to receive attention.

In particular, as shown in our earlier exploration in this field, $L^{2}$RIRNet [20] adopts a dual-branch architecture with pairwise degradation feature vector guidance, which models low-light degradation and rain degradation separately and verifies that task-specific architectures are necessary for this coupled restoration problem.

However, this data-driven degradation representation relies heavily on the training degradation distribution, and it lacks explicit and stable structural guidance under strongly coupled low-light and rain corruptions. Meanwhile, it does not fully resolve the conflicting feature responses caused by two degradations in the shared feature space, which limits the restoration performance under severe coupled-degradation scenarios. Motivated by these limitations, this work explores a new technical route from the perspectives of robust prior guidance and adaptive feature interaction.

Nevertheless, the problem remains challenging because the degradation is highly spatially variant: rain patterns are often buried in severely dark regions, yet become much more visible after illumination enhancement. Consequently, sequential pipelines or rigid decomposition strategies still struggle to remove rain consistently without sacrificing structural fidelity.

**Figure 1:**
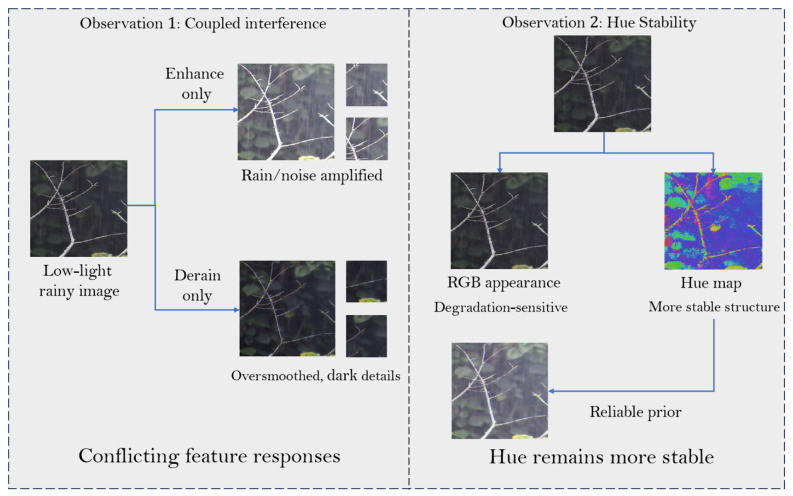
Motivation of the proposed DHP-Net. **Observation 1:** In low-light rainy scenes, brightness enhancement and deraining interfere with each other: enhancement tends to amplify hidden rain streaks and noise, while deraining may suppress already weak scene structures. **Observation 2:** Compared with Red, Green, Blue (RGB) appearance, the hue component in Hue, Saturation, Value (HSV) space remains more stable under degradation and preserves clearer scene layout and object boundaries, making it a reliable prior for restoration.

As illustrated in Figure 1, two observations motivate our design. First, under a shared parameter space, low-light enhancement and deraining do not impose fully consistent feature preferences: features useful for brightness recovery may amplify rain responses, whereas features emphasizing rain suppression may weaken fine scene structures. Instead of introducing separate optimization routes for each degradation, we seek to reorganize and recalibrate features within the shared representation space so that intrinsic image content is preserved while degradation interference is reduced. Second, not all image cues are equally sensitive to coupled degradations. Compared with RGB appearance, the hue component in HSV space remains more stable under degradation and preserves clearer scene layout and object boundaries. These observations suggest a restoration framework that combines a degradation-robust hue prior with adaptive feature perturbation and interaction.

Based on these observations, we propose **DHP-Net**, a **D**egradation-robust **H**ue **P**rior Network for low-light rainy image restoration. DHP-Net is a single-stage end-to-end framework with dual-branch synergistic guidance:

- **Hue Prior Extraction Network**: This branch exploits the degradation robustness of the hue channel to extract multi-scale color priors from the degraded input. These priors preserve stable background and structural cues and provide explicit hierarchical guidance during restoration.
- **Transformer-based Restoration Network with Adaptive Feature Modulation**: The main branch adopts an efficient Transformer backbone and introduces a **C**hannel-**a**daptive **A**ttention **P**erturbation **M**odule (CAAPM). The key idea is to perturb the intermediate feature organization before attention-based interaction, so that degradation-sensitive responses can be redistributed and selectively recalibrated in the shared feature space. By combining channel shuffling with perturbed cross-channel attention, the module promotes adaptive interaction among detail features, contextual features, and multi-scale hue priors, enabling more effective removal of coupled degradations. The entire network is optimized end-to-end with pixel, perceptual, and frequency-domain supervision.

The main contributions of this paper are summarized as follows:

1. We propose DHP-Net, a single-stage end-to-end framework for low-light rainy image restoration that jointly models brightness recovery and rain removal through synergistic prior guidance and adaptive feature modulation.
2. We design a hue prior extraction branch that produces multi-scale degradation-robust color features and injects them hierarchically into the restoration process for stable color recovery and structure preservation.
3. We integrate a channel-adaptive attention perturbation mechanism into the Transformer backbone to enhance cross-feature interaction and improve restoration under coupled low-light and rain degradations.

## 2. Related Work

### 2.1. Single-Degradation Image Restoration

Most existing restoration methods are designed for a single degradation, such as deraining, low-light enhancement, deblurring, denoising, snow removal [21], and super-resolution.

Generally, CNN-based methods excel at capturing local texture details but are limited in modeling long-range contextual dependencies, whereas Transformer-based architectures achieve stronger global receptive fields but typically incur higher computational costs.

Dedicated architectures have also been developed for document shadow removal [22]. Earlier Convolutional Neural Network (CNN)-based methods established strong local modeling ability across these tasks [4,7,16,23], while more recent Transformer-based models improved global context aggregation and long-range dependency modeling. In particular, Restormer [13] demonstrated that efficient channel-wise attention and hierarchical encoder–decoder design can achieve strong performance on high-resolution restoration, and has since become a widely adopted backbone. For rain removal, recent work has extended beyond pure rain streak removal to address raindrops, diverse rain patterns, and physical degradation modeling: CSUD [6] exploits channel consistency for unsupervised deraining, CLIP-RPN [24] adaptively routes rainy inputs to specialized subnetworks via vision–language perception, RIADNet [25] jointly removes raindrops and rain streaks, and SD-PSFNet [26] incorporates learnable point spread functions to model rain streak optics. For low-light enhancement, HVI [12] introduces a novel color space to reduce color and brightness artifacts. For image deblurring, MDT [27] leverages motion vector decomposition to handle complex rotational blur, EVSSM [28] employs efficient visual state-space models for high-resolution deblurring, stochastic refinement strategies have been explored for uncertain degradations [29], and ViT-FFT-ReLU [30] integrates frequency-domain sparsity with Transformer-based attention, and stochastic refinement strategies [29] further improve perceptual deblurring quality. For super-resolution, KAST [31] adapts Swin Transformer with kernel-aware degradation modeling for blind SR, KernFusNet [32] fuses implicit kernel estimation with detail refinement, DAFST [33] employs degradation-aware frequency separation to recover high-frequency structures, and Real-ESRGAN [34] provides a representative synthesis-training paradigm for real-world super-resolution. Despite their success, these methods are optimized for their target isolated degradations and lack explicit consideration of mutual interference between coexisting corruptions, so they usually generalize poorly to coupled low-light rainy scenes.

### 2.2. Mixed-Degradation Restoration

Mixed-degradation restoration addresses images corrupted by multiple degradation factors simultaneously, such as noise, blur, rain, haze, and low illumination. Compared with single-degradation restoration, this setting is substantially more challenging because different degradations may overlap in the same spatial regions and induce conflicting restoration objectives. Early practical solutions often relied on cascaded pipelines that sequentially apply single-task models. These pipelines are straightforward to build and can reuse well-trained single-task models, but they inevitably suffer from error accumulation across stages and cannot effectively handle the mutual interference between tasks. This limitation has motivated the development of unified all-in-one or mixed-degradation-restoration frameworks that learn shared representations for multiple corruption types. PyDiff [35] improves feature modulation and generative restoration for diverse degradations, and latent prior encoding has been introduced for degradation-aware all-in-one restoration [36]; recent work further explores compositional degradation in UAV imagery [37] and multi-task architectures handling low-light, noise, blur, and low-resolution jointly [38].

However, most general mixed-degradation frameworks treat different corruption types equally and do not model the strong coupling relationship between low-light degradation and rain streaks, which limits their performance upper bound on this specific task.

Within this broader setting, low-light rainy image restoration represents a particularly difficult mixed-degradation problem because illumination degradation and rain corruption are not simply coexisting artifacts but strongly coupled factors. A common baseline is still to cascade single-task models, yet sequential processing usually fails in practice: enhancement may amplify hidden rain or noise, while deraining or deblurring may further suppress weak scene structures under poor illumination [38]. To address these issues, recent work has designed dedicated joint-restoration frameworks.

Specifically, our previous work $L^{2}$RIRNet [20] handles the joint task through a dual-branch framework with separate degradation feature modeling. In contrast, the proposed DHP-Net differs from it in three core aspects:

- *Guidance mechanism*: $L^{2}$RIRNet relies on data-driven learned pairwise degradation features for guidance, while DHP-Net introduces a hue prior derived from color space properties, which provides more stable and degradation-robust structural and color cues without depending on training degradation distributions.
- *Architecture design*: $L^{2}$RIRNet uses a dual-branch structure to model two degradations separately, while DHP-Net builds a single-stage unified Transformer backbone and resolves conflicting feature responses in the shared feature space via the Channel-adaptive Attention Perturbation Module, achieving more efficient joint optimization.
- *Supervision strategy*: On the basis of pixel and perceptual loss, DHP-Net further introduces a stationary wavelet frequency-domain loss to impose targeted constraints on the frequency components most affected by coupled degradation.

These design differences enable DHP-Net to achieve better restoration performance under coupled low-light and rain conditions. For low-light rainy restoration, MR-SDformer [39] and NDMamba [40] model cross-degradation interactions via Retinex-based decomposition and dual-prior state-space mechanisms, while benchmarks such as the one proposed in [41] provide physically plausible evaluation data. For low-light deblurring, DarkIR [42] and JUDE [43] propose efficient multi-task networks and physics-inspired unrolling architectures; DAP-LED [44] leverages vision–language priors for joint enhancement and deblurring, and zero-reference frameworks with vision language model-derived modulation have also been developed [45]. For super-resolution with additional degradations, FMA-Net [46] jointly addresses video SR and deblurring, TAFCNet [47] couples motion deblurring with SR, and DenoSR [48] tackles SR under noise. These studies indicate that low-light rainy restoration should be viewed as a specialized mixed-degradation problem requiring dedicated modeling of cross-degradation interactions.

In addition to the above frameworks, recent studies have further explored novel mixed-degradation modeling paradigms, including mixture-of-experts based dynamic degradation routing, diffusion-driven generative restoration, and state-space model-based long-range dependency modeling. While these methods achieve competitive performance on general mixed-degradation benchmarks, most of them are optimized for combinations of noise, blur, and low-resolution, and they do not take into account the strong coupling between illumination attenuation and rain streak corruption. Therefore, they lack targeted design for the low-light rainy restoration task, which is the core focus of this work.

### 2.3. Prior-Guided and Frequency-Aware Restoration

Another important line of research improves restoration by introducing informative priors or auxiliary feature modulation mechanisms. Internal priors derived from the degraded input are particularly attractive because they are easy to obtain and often physically meaningful. Self-supervised visual priors from models like DINOv2 have been integrated into restoration for ambient light normalization [49], while frameworks such as FiRe [50] expand PnP priors to general restoration models by treating smooth images as fixed points of degradation-restoration composition. In parallel, restoration models have increasingly adopted feature modulation strategies to better adapt intermediate representations to different degradations. PromptIR [14] and PromptRestorer [17] use prompt-like conditioning, and PSAIR [51] leverages semantic priors from pretrained segmentation models to adaptively modulate degraded features via learnable visual prompts.

Generally, external pretrained priors can provide rich semantic information but usually bring considerable computational overhead and may face domain adaptation problems in specific low-level tasks. Internal image priors are more lightweight and domain adaptive, but many existing internal priors are still sensitive to strong coupled degradations.

Frequency-domain modeling provides another complementary direction. UHD-Processor [52] employs progressive frequency learning with degradation-aware prompts for all-in-one UHD restoration, and dynamic frequency-aware Transformer architectures have also been proposed for general restoration tasks [53]. SPJFNet [54] introduces a Self-Mining Guidance Module to generate lightweight endogenous priors and performs joint wavelet–Fourier frequency enhancement. AdaIR [55] adaptively mines and modulates frequency sub-bands based on input degradation type, and wavelet-based deraining methods such as WD-MFSU [56] and FRAMT [57] integrate frequency-domain modulation with spatial features to preserve structures and suppress rain artifacts.

Nevertheless, most existing frequency-aware designs are developed for single-degradation scenarios and do not fully consider the complex frequency component interaction caused by coupled low-light and rain corruptions.

However, existing degradation-aware restoration frameworks still have limitations for strongly coupled low-light rainy scenes. Prompt-based methods such as PromptIR [14] and AdaIR [55] mainly rely on external conditioning or adaptive frequency mining to adapt to independent degradation types, but they fail to explicitly model the strong mutual interference between brightness enhancement and rain removal. General feature perturbation strategies can improve feature diversity for diverse degradations, yet they lack stable structural prior guidance under severe coupled corruption, and their perturbation design is not tailored to the feature entanglement caused by coexisting low-light attenuation and rain streak distortion.

Together, these studies indicate that robust priors, adaptive feature modulation, and frequency-aware constraints are effective tools for handling complex restoration problems. Inspired by these findings, this work combines a degradation-robust internal hue prior with channel-adaptive feature perturbation and frequency-domain supervision to address the coupled low-light rainy restoration task.

To further clarify the novelty of the proposed method and its fundamental differences from existing restoration frameworks, we conduct a systematic comparative analysis of representative approaches from three core dimensions: prior source, feature modulation strategy, and architecture paradigm.

In terms of prior design, existing restoration methods mainly adopt three technical routes. General mixed-degradation methods such as PromptIR rely on external learnable degradation prompts such as conditioning, and AdaIR introduces frequency-domain sub-band priors to adapt to diverse degradation types. Such priors are designed for general mixed-degradation scenarios and lack targeted adaptation to the strong coupling characteristics of low-light attenuation and rain streak distortion. The dedicated low-light rainy method $L^{2}$RIRNet adopts data-driven learned degradation features for guidance, but its feature representation highly depends on the training degradation distribution, and the structural guidance stability is insufficient under severe coupled degradation. In contrast, DHP-Net introduces an internal hue prior derived from the inherent properties of HSV color space. This prior does not rely on training data distribution nor extra pretrained models, and maintains more stable scene layout and boundary cues under coupled degradation, providing reliable explicit structural guidance for restoration.

In terms of feature modulation strategy, mainstream restoration methods mostly adopt channel-wise attention weighting, degradation-aware routing or frequency sub-band recalibration to adapt intermediate features. These strategies essentially adjust the importance weight of different feature channels on the original feature space, which cannot effectively resolve the conflicting feature responses caused by the coexistence of low-light enhancement and rain removal demands. The proposed channel-adaptive attention perturbation adopts a different technical logic: it first reorganizes the channel organization of intermediate features through input-adaptive shuffling, and then performs cross-view attention recalibration between the perturbed feature view and the original view. By reshaping the organization of the shared feature space itself, it directly alleviates the feature entanglement caused by coupled degradation, which is more targeted for the low-light rainy restoration task.

In terms of architecture paradigm, existing dedicated methods for coupled low-light rainy restoration mostly adopt a dual-branch architecture that models low-light degradation and rain degradation separately, which inevitably brings computational redundancy and inter-stage error accumulation. DHP-Net adopts a single-stage unified Transformer backbone, and realizes the joint optimization of brightness recovery and rain removal within a single network through hierarchical prior injection and built-in feature perturbation modules. This design avoids the defects of multi-branch or cascaded pipelines while ensuring restoration performance, and has higher computational efficiency and deployment friendliness.

The above differences jointly constitute the core novelty of this work: starting from the two perspectives of robust prior guidance and adaptive feature interaction, we construct a targeted single-stage solution for the strongly coupled low-light rainy degradation, which effectively makes up for the shortcomings of existing general restoration frameworks and dual-branch dedicated methods.

Beyond the above frequency and prior-driven restoration frameworks, Blind Image Quality Assessment (BIQA) tailored for low-light scenarios provides valuable complementary perspectives for quantitative evaluation of restoration performance. Traditional full-reference metrics such as PSNR and SSIM rely on paired clean ground-truth images, which are unavailable for real-world unpaired low-light rainy data, limiting their applicability in practical degradation scenarios.

Recent advances in low-light BIQA have developed human perception-aligned metrics for reference-free quality measurement. As representative works, Wang et al. [58] propose a blind multimodal quality assessment framework for low-light images via multi-domain feature fusion, and Wang et al. [59] design a visibility perception-guided blind quality indicator specifically for in-the-wild low-light scenes. Both studies demonstrate that perceptual blind metrics can effectively characterize visibility degradation, color distortion and noise artifacts without ground-truth references. These BIQA paradigms provide a solid theoretical basis for our multi-dimensional no-reference evaluation system, and complement pixel-level full-reference metrics to enable more comprehensive, human perception-consistent performance assessment of low-light rainy image restoration methods on real-world datasets.

## 3. Method

### 3.1. Overview

The goal of low-light rainy image restoration is to recover a clean image from an observation degraded by both insufficient illumination and rain streaks. This setting is challenging because scene structures and degradation cues are highly entangled in the same feature space: brightness enhancement may reveal hidden rain artifacts, whereas aggressive deraining may suppress already weak structures. Moreover, under a shared backbone, different degradations can induce inconsistent intermediate responses, making it difficult to preserve intrinsic image content while suppressing degradation-specific interference.

**Figure 2:**
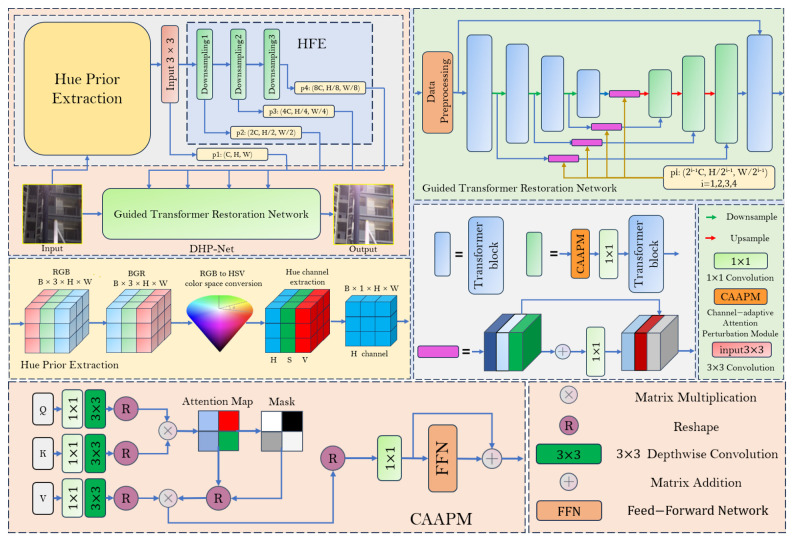
Overview of the proposed DHP-Net. The framework contains three core components: (1) a prior encoder with Hierarchical Feature Extraction (HFE), which extracts multi-scale degradation-robust hue priors from the degraded input; (2) a Transformer-based encoder–decoder restoration network, which progressively reconstructs the clean image under multi-scale prior guidance; and (3) a shuffle-based perturbation module, which enhances cross-feature interaction for coupled low-light and rain degradation removal. The abbreviation FFN in the figure denotes Feed-Forward Network. All operational symbols are defined in the in-figure legend, and color shading is used only to distinguish functional branches.

To decouple this, we propose DHP-Net, a single-stage framework that combines degradation-robust prior extraction with adaptive Transformer-based restoration. As shown in Figure 2, the framework consists of three components: (1) a prior encoder that extracts multi-scale hue priors from the degraded input; (2) a Transformer encoder–decoder restoration network that progressively reconstructs the clean image under hierarchical prior guidance; and (3) a shuffle-based feature perturbation module that reorganizes intermediate responses and strengthens cross-feature interaction for coupled-degradation modeling.

Given an input image *I*, we first convert it into HSV space and extract the hue channel as a degradation-robust source of structural and color information. The prior encoder maps this cue into hierarchical prior features at four scales. In parallel, the RGB image is processed by a four-stage Transformer encoder. At the encoder stages and the latent bottleneck, we insert the Channel-adaptive Attention Perturbation Module (CAAPM) immediately before channel interaction, so that the incoming restoration features are first reorganized and then fed into attention-based recalibration. In other words, CAAPM is not an external side branch; it is an internal feature-conditioning block placed inside the main restoration stream, where low-light and rain responses are most entangled. The decoder progressively upsamples the latent representation and fuses encoder features with aligned multi-scale hue priors to reconstruct the restored image.

Notably, different from existing degradation-aware frameworks that depend on external prompt conditioning or general feature perturbation, our method leverages an internal degradation-robust hue prior for hierarchical guidance, and designs a task-specific channel-adaptive perturbation mechanism to resolve coupled feature conflicts. This design is more targeted for the strong coupling between low-light degradation and rain streak corruption.

The full network is trained end-to-end using pixel-level, perceptual, and frequency-domain supervision.

### 3.2. Hue Prior Extraction Network

The hue prior branch is introduced to provide a stable reference for restoration under coupled degradations. In low-light rainy images, RGB intensities are easily distorted by under-exposure, rain accumulation, and subsequent enhancement, whereas the hue component remains relatively more stable and therefore preserves useful background cues [23,60]. Based on this observation, we convert the degraded input into HSV space and feed the hue channel into a lightweight prior encoder. The encoder first embeds the single-channel hue map into a feature space and then progressively extracts hierarchical representations through a multi-scale convolutional pathway. In this way, the branch produces a set of prior features that capture complementary cues from local structures to coarse scene layout.

**Figure 3:**
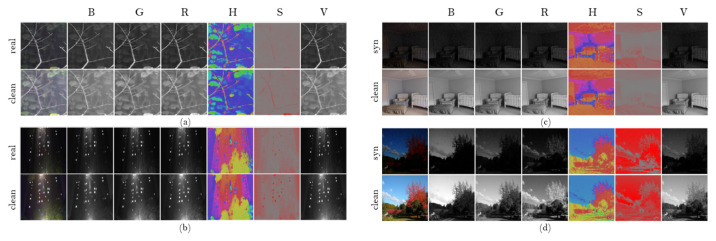
Channel-wise comparison between degraded and clean images in RGB and HSV spaces. The labels (**a**–**d**) are shown only for visual illustration and are not described separately. Across both real and synthetic examples, the RGB channels and the value component vary substantially under degradation, while the hue channel remains comparatively stable in scene layout, object boundaries, and region-level color organization. This observation supports the use of hue as a degradation-robust prior for low-light rainy restoration.

This observation is further illustrated in Figure 3, which contains four sub-figures organized into two groups: a real-world sample group and a synthetic sample group. Each group includes a degraded RGB image and its corresponding hue map, forming four parts (a)–(d) in total, which compares RGB and HSV channels under both real and synthetic degradations. Although the degraded and clean images exhibit large discrepancies in the RGB channels as well as in the value component, the hue maps remain noticeably more consistent in scene layout, object boundaries, and region-level color organization.

To quantitatively validate this observation, we perform rigorous statistical analysis on all 800 paired samples from the LLR synthetic test set. We evaluate channel-wise consistency between degraded and clean images via three complementary metrics: structural similarity (SSIM), mutual information (MI), and entropy stability. All metric results are reported as mean ± standard deviation, and one-sided paired *t*-tests with Holm family-wise correction are adopted to verify statistical significance.

Quantitatively, the hue (H) channel yields an SSIM of 0.7714±0.1369, substantially outperforming the R (0.4333±0.1442), G (0.4207±0.1469), B (0.3924±0.1482), S (0.6166±0.1421), and V (0.4597±0.1410) channels. Pairwise Holm-corrected paired *t*-tests confirm that all inter-channel differences in SSIM are extremely statistically significant (all adjusted p<0.001). While the R, G, B and V channels exhibit higher mutual information values, such metric superiority only reflects global brightness correlation and cannot characterize the retention of spatial scene structures. By contrast, the markedly higher SSIM of the hue channel demonstrates its stronger capacity to preserve scene layouts and object contours under coupled degradations.

In terms of entropy stability, the hue channel attains a mean value of −0.3207±0.4228. Its entropy stability metric is notably closer to zero compared with the remaining channels: R (−1.1838±0.7436), G (−1.2436±0.7700), B (−1.4441±0.8500), S (−0.5071±0.6234) and V (−0.9971±0.6928). All pairwise differences between the H channel and other channels pass the significance test with all adjusted p<0.001, indicating that coupled degradations induce minimal information distortion and loss within the hue component. Collectively, these quantitative measurements and statistical significance tests deliver solid statistical evidence supporting the superior robustness of the hue channel against coupled image degradations.

The prior encoder is not used as an independent restoration stream. Instead, it serves as a guidance branch that continuously regularizes the feature learning process of the main network. Specifically, the prior features are aligned with the corresponding stages of the Transformer backbone and fused through residual feature projection. This design allows the model to inject degradation-robust color and structure information directly into the evolving representation without overwriting the original image-dependent features. As the network goes deeper, the prior branch provides increasingly abstract background guidance, which helps the model distinguish genuine scene content from degradation-induced responses. Compared with directly concatenating hue to the input, this hierarchical design yields a more effective use of prior information because the guidance is delivered where the degradation modeling actually occurs.

The detailed channel-wise statistical results are summarized in Table 1.

### 3.3. Restoration Network

The main restoration branch follows a hierarchical Transformer encoder–decoder architecture [1]. Starting from the degraded RGB image, the encoder progressively aggregates local structures and long-range contextual dependencies, while the decoder reconstructs the clean image through multi-level skip connections. Different from a standard Restormer-style pipeline, our backbone is guided by hue priors during feature extraction and is further equipped with a perturbation-based attention mechanism that explicitly targets the interference caused by coupled degradations.

The core idea of the restoration network is to avoid directly performing attention over highly entangled features. In low-light rainy scenes, the same intermediate channel may simultaneously respond to background edges, brightness deficiency, rain streaks, and amplified noise. If such features are directly aggregated, the resulting attention tends to reinforce degradation correlations rather than suppress them. To mitigate this issue, we introduce the Channel-adaptive Attention Perturbation Module (CAAPM), which perturbs the channel organization before attention aggregation and then performs cross-feature recalibration in the perturbed space. The design of the feature perturbation paradigm is inspired by the degradation-aware feature modulation framework for general mixed-degradation image restoration, and we adapt it to the coupled low-light rainy scenario by adjusting the channel routing strategy and integrating it with the multi-scale hue prior fusion pipeline.

Different from a standard channel shuffle that applies a fixed hand-crafted permutation to all inputs, CAAPM uses shuffle as a lightweight mixing primitive and couples it with input-adaptive routing. The specific implementation process is as follows:

1. **Global context encoding and routing code generation**: Given an input feature tensor $F\in R^{C\times H\times W}$, we first aggregate global spatial information through global average pooling to obtain a channel-wise context vector. A lightweight two-layer MLP branch then maps this context vector into a set of channel importance weights, namely the routing code $r\in R^{C}$. This routing code is dynamically generated according to the input degradation state, so the channel perturbation pattern varies with the content and degradation severity of the input image.

2. **Adaptive channel regrouping**: Based on the predicted routing code, we sort the channels by importance and perform group-wise channel shuffling on the reordered channels. In our implementation, we set eight channel groups by default. This operation reorganizes the original channel arrangement and forms a perturbed feature view $F_{p}$, which breaks the original coupling relationship between degradation responses and scene content responses in the channel dimension.

3. **Cross-view cross-channel attention**: We retain the original feature F as the reference view, and compute cross-channel attention between the reference view and the perturbed view $F_{p}$. Specifically, we take the channel descriptors of the perturbed view as queries and the channel descriptors of the original view as keys and values, so that the model can learn reliable channel dependencies from the reorganized feature space, and selectively activate channels beneficial to scene restoration while suppressing channels dominated by rain streaks and low-light noise.

4. **Residual output**: The recalibrated feature map is fused with the original input feature through a residual connection to obtain the final output of the CAAPM module.

By reshaping the organization of the shared feature space rather than explicitly separating different degradations into independent branches, the proposed mechanism effectively alleviates the feature entanglement problem caused by coupled low-light and rain degradations.

The decoder progressively reconstructs the image from the latent representation using skip-connected encoder features. Since the encoder features have already been calibrated by prior fusion and channel-adaptive perturbation, the decoder can focus on recovering fine structures, contrast, and color consistency. A final refinement stage and residual output head predict the restored RGB image. Overall, the restoration branch combines hierarchical Transformer modeling, prior-guided encoder fusion, and perturbation-aware attention recalibration, resulting in a unified architecture tailored to the coupled low-light rainy restoration problem.

### 3.4. Training Objective

We optimize the entire framework using a composite objective that jointly constrains restoration fidelity, perceptual realism, and spectral consistency. The overall loss is defined as:

(1) $$L_{\mathrm{total}}=L_{L1}+\lambda_{1}L_{\mathrm{perc}}+\lambda_{2}L_{\mathrm{freq}},$$

where, $L_{L1}$ is the pixel reconstruction loss and $L_{\mathrm{perc}}$ is the Visual Geometry Group (VGG)-based perceptual loss, which is widely adopted to improve perceptual fidelity in image generation and restoration [61]. The coefficients $\lambda_{1}$ and $\lambda_{2}$ are scalar balancing weights that control the relative contributions of the perceptual and frequency-domain terms with respect to the pixel reconstruction loss. The third term, $L_{\mathrm{freq}}$, is an illumination-aware wavelet structure consistency loss. Instead of supervising the restored image only in the spatial domain, this term applies the Stationary Wavelet Transform (SWT) to the luminance component and penalizes the discrepancy between the restored and ground-truth sub-bands, following the broader practice of wavelet decomposition theory and frequency-aware restoration [56,62,63,64]. The motivation is that low-light rainy degradation affects different frequency components in different ways: low-frequency bands mainly govern global illumination and smooth background consistency, whereas high-frequency bands are more sensitive to rain streaks, noise, and edge details. Consequently, $L_{\mathrm{freq}}$ provides a targeted constraint on the frequency components that are most affected by coupled low-light and rain degradation.

Specifically, let $Y_{r}$ and $Y_{g}$ denote the luminance channels of the restored image and ground-truth image, respectively. We apply a one-level Stationary Wavelet Transform to obtain four sub-bands for each image:

(2) $$\mathrm{SWT}\left(Y\right)=\left\{Y^{LL},Y^{LH},Y^{HL},Y^{HH}\right\},$$

where the first letter represents the row-direction filtering and the second letter represents the column-direction filtering, with *L* standing for low-pass filtering and *H* standing for high-pass filtering.

Specifically:

- LL: low-frequency approximation sub-band, which carries global illumination and smooth background information;
- LH: horizontal low-pass/vertical high-pass sub-band, which mainly captures vertical edge structures;
- HL: horizontal high-pass/vertical low-pass sub-band, which mainly captures horizontal edge structures;
- HH: dual high-pass sub-band, which corresponds to diagonal textures, rain streaks, and high-frequency noise.

Based on this decomposition, the frequency-domain loss is defined as:

(3) $$L_{\mathrm{freq}}=∥Y_{r}^{LL}-Y_{g}^{LL}{∥}_{1}+∥Y_{r}^{LH}-Y_{g}^{LH}{∥}_{1}+∥Y_{r}^{HL}-Y_{g}^{HL}{∥}_{1}+∥Y_{r}^{HH}-Y_{g}^{HH}{∥}_{1}.$$

We compute this loss on the luminance channel rather than directly on RGB because the coupled degradation is most prominently reflected in brightness attenuation and rain-induced structural corruption. In this decomposition, the LL term constrains global illumination recovery and smooth background transitions, while the LH, HL, and HH terms explicitly penalize residual rain streaks, halo artifacts, and blurred edges. In this way, $L_{\mathrm{freq}}$ complements the spatial-domain reconstruction loss and the perceptual loss by imposing a more targeted constraint on the frequency components that are most sensitive to low-light rainy degradation. In all experiments, the overall contribution of this frequency-domain term is controlled by $\lambda_{2}$, which is set to 0.15 as described in the implementation details and validated in the ablation study.

Compared with plain pixel supervision, this frequency-aware objective provides a stronger constraint on structure recovery under coupled degradations. It is particularly useful in dark rainy scenes where brightness correction can easily introduce spurious high-frequency patterns. In our implementation, $L_{\mathrm{freq}}$ is computed on the luminance channel to make the supervision more sensitive to illumination recovery, while still remaining complementary to the RGB-based pixel and perceptual losses.

## 4. Experiments

### 4.1. Dataset

To train and evaluate DHP-Net, we use the Low-Light Rain (LLR) dataset introduced in $L^{2}$RIRNet [20], with the official dataset and code repository available at [65]. The LLR dataset is specifically designed for the joint restoration of low-light degradation and rain corruption, which makes it more suitable for our task than conventional single-degradation benchmarks. It contains 800 synthetic paired samples, where each degraded rainy low-light image is paired with a corresponding clean reference image for supervised training and full-reference evaluation. In addition, the dataset provides 42 real-world rainy low-light images without ground-truth references, which are mainly used for qualitative comparison and no-reference perceptual evaluation. The scenes cover diverse objects, structures, and illumination conditions, including challenging regions with weak visibility, rain interference, and fine structural details. Therefore, LLR offers a representative and task-specific benchmark for evaluating restoration performance under coupled illumination degradation and rain streak corruption.

### 4.2. Experimental Setup

#### 4.2.1. Implementation Details

Our restoration network adopts a hierarchical U-shaped Transformer encoder–decoder architecture, namely DHP-Net, which is guided by a degradation-robust hue prior and equipped with channel-adaptive feature perturbation. The backbone contains four encoder stages and four corresponding decoder stages, with channel dimensions increasing progressively from shallow to deep layers. The hue prior branch is a lightweight four-stage convolutional encoder, and its multi-scale features are aligned with the main restoration stages through 1×1 projection layers. The CAAPM module is inserted into the encoder and latent stages; in our implementation, channel perturbation is performed with eight-group shuffling, and the cross-attention branch uses a reduction ratio of four for channel interaction.

To optimize the proposed model, we employ the Adaptive Moment Estimation (Adam) optimizer with $\beta_{1}=0.9$, $\beta_{2}=0.999$, and $\epsilon =10^{-8}$. The initial learning rate is set to $2\times 10^{-4}$ and gradually reduced to $10^{-6}$ via a cosine annealing scheduler [66]. All experiments are implemented in PyTorch v2.1.0 (Meta Platforms, Inc., Menlo Park, CA, USA; available at: https://pytorch.org/; accessed on 20 May 2026) with a single-stage end-to-end training strategy. The training objective is composed of the L1 reconstruction loss, the VGG perceptual loss, and the proposed frequency-domain loss, with balancing weights set to $\lambda_{1}=0.05$ and $\lambda_{2}=0.15$. All models are trained on a single NVIDIA GeForce RTX 3090 GPU (NVIDIA Corporation, Santa Clara, CA, USA). The batch size and training patch size are configured via the ‘training.yml’ file, with a default batch size of 1 and a patch size of 64×64.

#### 4.2.2. Evaluation Metrics

We adopt two widely used full-reference metrics for quantitative evaluation on the synthetic test set of the LLR dataset: Peak Signal-to-Noise Ratio (PSNR) and Structural Similarity Index Measure (SSIM). PSNR is used to evaluate pixel-level fidelity between the restored image and the ground-truth clear image, while SSIM is adopted to assess the structural consistency and perceptual quality of the restoration results.

In addition, we introduce the Learned Perceptual Image Patch Similarity (LPIPS) as a supplementary full-reference metric. It measures the perceptual distance between restored images and ground-truth references based on deep feature representations, which is more consistent with human visual perception. A lower LPIPS value indicates better perceptual quality and higher visual fidelity.

Both metrics are calculated using our official implementation with consistent parameter settings for all compared methods.

For no-reference perceptual evaluation on real-world images, we adopt the Natural Image Quality Evaluator (NIQE) in the original experiment, where lower values indicate better restoration quality.

To further establish a comprehensive no-reference evaluation system, we supplement four additional metrics from different perspectives:

- Blind/Referenceless Image Spatial Quality Evaluator (BRISQUE): a classical spatial-domain blind image quality metric that quantifies natural scene statistical distortion. Lower values indicate better overall image quality.
- Perception-based Image Quality Evaluator (PIQE): a no-reference metric focusing on local block distortion and structural degradation, with lower values indicating fewer visible artifacts.
- Lightness Order Error (LOE): a task-specific metric for low-light enhancement, which evaluates the consistency of relative brightness order before and after restoration. Lower LOE values indicate more natural illumination preservation and less brightness distortion.
- Information Entropy: a statistical metric that reflects the richness of image details and information content. Higher entropy values indicate more abundant texture and grayscale information in the restored image.

Together with NIQE, these metrics form a multi-dimensional evaluation system covering general image quality, illumination fidelity, and information richness. In addition, we include two deep learning-based no-reference metrics as supplementary evaluation: Multi-Scale Image Quality (MUSIQ) and CLIP-based Image Quality Assessment (CLIP-IQA), both of which evaluate perceptual naturalness from the perspective of deep feature semantics.

Aligned with core insights from recent low-light blind image quality studies [58,59], we incorporate no-reference perceptual quality metrics into our evaluation protocol to mitigate over-reliance on pixel-wise full-reference indicators, achieving more human perception-consistent quantitative comparison on real-world low-light rainy data.

### 4.3. Comparison on the LLR Dataset

We quantitatively evaluate the restoration performance of the proposed DHP-Net on the synthetic test images from the LLR dataset. Since the joint low-light rainy image restoration task is rarely explored, there are no dedicated open-source methods for direct comparison. To conduct a comprehensive and fair evaluation, we select representative state-of-the-art methods and construct two types of baselines: cascaded methods and retrained methods.

#### 4.3.1. Compared Methods

For fair comparison, we follow $L^{2}$RIRNet [20] in selecting both cascaded methods and retrained methods as baselines. Specifically, the cascaded setting combines representative low-light enhancement and image deraining models in a sequential pipeline, while the retrained setting contains representative general restoration networks retrained on the LLR dataset under the same protocol. We also follow $L^{2}$RIRNet [20] in reporting the corresponding quantitative results for these compared methods. To further verify the competitiveness of the proposed method against the latest state-of-the-art mixed-degradation-restoration approaches, we supplement two representative latest methods, AdaIR [55] and PromptIR [14], as additional baselines. Both methods are retrained on the LLR training set under exactly the same training protocol as DHP-Net to ensure fair comparison, and we conduct a full-dimensional quantitative comparison on the synthetic test set.

It should be clarified that the current benchmark does not cover all recently published mixed-degradation-restoration methods, mainly due to three constraints: first, most generic mixed-degradation methods are not designed for the coupled low-light rainy scenario, with inconsistent degradation assumptions from our task setting; second, some recent works have not released official source code or task-specific training configurations, and manual reimplementation may introduce performance bias that undermines comparison fairness; third, part of the frameworks require additional input conditions (e.g., degradation labels, prompt embeddings) that are incompatible with the single-image input setting of the LLR benchmark. We will continuously expand the comparison scope with more state-of-the-art methods in subsequent research on this specialized task.

#### 4.3.2. Quantitative Evaluation

For the synthetic subset, we compute the average PSNR and SSIM over all 800 test images. As reported in Table 2, DHP-Net achieves the best overall performance, reaching 33.14 dB PSNR and 0.9252 SSIM. Among the retrained baselines, the strongest competitor is $L^{2}$RIRNet-Trans* [20], which obtains 32.53 dB and 0.9231 SSIM. Our method surpasses it by 0.61 dB in PSNR and 0.0021 in SSIM. DHP-Net also outperforms other strong restoration baselines, including DRSFormer* [1] by 1.56 dB/0.0070 SSIM and Restormer* [13] by 1.95 dB/0.0078 SSIM. The gap is even larger when compared with cascaded methods. The best cascaded baseline, SCI ⇒ MIRNet, achieves only 20.71 dB PSNR and 0.7186 SSIM, which is 12.43 dB and 0.2066 lower than DHP-Net. This substantial margin confirms that sequentially combining low-light enhancement and deraining models is insufficient for the coupled-degradation setting.

To characterize the performance difference between DHP-Net and the latest general mixed-degradation-restoration methods, we conduct a full-metric comparative evaluation on the synthetic test set with AdaIR and PromptIR, covering pixel fidelity, perceptual quality, illumination consistency and information richness. The detailed results are summarized in Table 3.

Experimental results show that DHP-Net achieves consistent advantages on most core metrics. Specifically, DHP-Net leads by 2.32 dB and 2.30 dB in PSNR compared with AdaIR and PromptIR, respectively, and achieves the lowest LPIPS value, which verifies that the dedicated coupled-degradation modeling brings significant improvement in both pixel-level reconstruction accuracy and perceptual visual quality. In terms of no-reference naturalness metrics, the three methods have their own strengths on different statistical indicators, while DHP-Net achieves the best performance on MUSIQ and CLIP-IQA which are more aligned with human semantic perception. This set of comparative results fully demonstrates that compared with general-purpose mixed-degradation-restoration frameworks, the task-specific design of DHP-Net has clear performance advantages on the low-light rainy coupled restoration task.

For the real-world subset, we evaluate the no-reference perceptual quality using NIQE, where lower values indicate better restoration quality. As shown in Table 4, the degraded input obtains a NIQE score of 16.93. Existing restoration methods reduce this value to different extents, with MIRNet* and NAFNet* achieving 14.31 and 14.65, respectively, while $L^{2}$RIRNet-Trans* reaches 14.17. Our DHP-Net further improves the NIQE score to **13.42**, outperforming all compared methods on real-world low-light rainy images. Together, the results in Table 2 and Table 4 demonstrate that the proposed degradation-robust hue prior and perturbation-aware restoration strategy generalize effectively from synthetic supervision to real-world degraded scenes.

To comprehensively evaluate the restoration performance of DHP-Net from multiple dimensions, we conduct a full-dimensional comparative evaluation with three representative competing methods on the synthetic test set, covering pixel fidelity, perceptual similarity, general image quality, illumination naturalness, and information richness. The detailed results are summarized in Table 5.

Experimental results show that DHP-Net maintains the optimal pixel-level reconstruction accuracy, with the highest PSNR and SSIM among all compared methods. For full-reference perceptual quality, DHP-Net achieves the lowest LPIPS value, which is significantly better than the other three baselines, verifying that the dedicated coupled-degradation modeling effectively improves perceptual visual fidelity.

For no-reference naturalness metrics, different methods exhibit respective advantages on different statistical indicators: $L^{2}$RIRNet-Trans* achieves better scores on BRISQUE, NIQE and PIQE, while DHP-Net maintains competitive overall performance while ensuring optimal structural fidelity. It should be noted that the LOE metric adopts inconsistent calculation scales between methods (DHP-Net uses normalized 0-1 values, while other methods use raw pixel-count values), so direct horizontal comparison is not performed.

For semantic perceptual metrics, DHP-Net and $L^{2}$RIRNet-Trans* both achieve superior CLIP-IQA scores, significantly outperforming the two general restoration baselines. The Information Entropy values of all methods are close, indicating that they can all effectively preserve image detail information. For the real-world test set, we also report the full-dimensional no-reference results of DHP-Net in the lower half of Table 5 as a reference for real-scenario performance.

To further verify the generalization capability of the proposed method across different rain patterns and scene distributions, we construct two extended test sets by incorporating unseen samples from the public rainy benchmarks Rain100L (light rain) and Rain100H (heavy rain). Each extended set consists of 800 images from the original LLR test set plus 40 unseen samples from the corresponding public benchmark. All models are trained exclusively on the original LLR training set, with no public benchmark samples included in training, to ensure a fair and reliable generalization evaluation.

The cross-set comparison results are summarized in Table 6. DHP-Net maintains stable restoration performance on both extended test sets. Even when heavy-rain samples with distinct degradation distributions are added, the performance drop remains moderate across all evaluation aspects, including pixel-level fidelity, perceptual naturalness, and illumination consistency. This demonstrates that the proposed degradation-robust hue prior and channel-adaptive perturbation mechanism can effectively adapt to rain streaks of different densities and unseen scene contents, verifying the strong cross-dataset generalization ability of our method.

And to clarify the computational efficiency and deployment feasibility of the proposed method, we conduct a detailed complexity analysis covering floating-point operations (FLOPs), inference latency, throughput, and peak GPU memory consumption at two common input resolutions. The detailed statistics are summarized in Table 7.

Benefiting from the single-stage end-to-end architecture design, DHP-Net achieves a competitive balance between restoration performance and computational overhead. At 256 × 256 resolution, the model runs at 15.73 Frames Per Second (FPS) with 743 MB peak memory, which can satisfy the basic real-time processing requirements of common surveillance and assisted driving scenarios, and can be easily deployed on mainstream edge GPU platforms. For 512 × 512 high-resolution inputs, the inference latency is about 288 ms, which is fully applicable to offline image enhancement tasks such as monitoring data post-processing. Although the overall computational scale is comparable to other Transformer-based restoration methods, our method achieves more significant performance gains on the coupled low-light rainy restoration task. In practical deployment, the model can be further accelerated through weight quantization, operator fusion, and structured pruning, which has good scalability for real-world applications.

It should be noted that the current cross-dataset generalization evaluation is mainly focused on variations of rain degradation, since this work targets the specific coupled low-light rainy restoration task. To further comprehensively verify the generalization capability of the proposed framework, extending validation to pure low-light benchmarks (e.g., LOL dataset series) and other mixed-degradation scenarios with different degradation combinations will be an important research direction in subsequent work.

To further quantify the computational overhead of the proposed core CAAPM module, we conduct a dedicated complexity analysis at 256 × 256 input resolution. The detailed statistics are summarized in Table 8. The CAAPM module is deployed at three encoder stages and one latent bottleneck stage, with a total of 4.98 M parameters and 8.79 G FLOPs, accounting for 15.70% of the total model parameters and only 5.21% of the total FLOPs, respectively. Combined with the ablation results, the CAAPM module achieves a 2.23 dB PSNR improvement with less than 6% additional computational cost, demonstrating an excellent performance–efficiency trade-off. The low computational proportion also verifies that the channel-adaptive perturbation design will not bring excessive inference burden to the overall framework.

#### 4.3.3. Qualitative Evaluation

Since no paired ground truth is available for real-world low-light rainy images, we conduct qualitative comparison via visual effect and no-reference quantitative metrics to verify the generalization ability of the proposed method.

**Figure 4:**
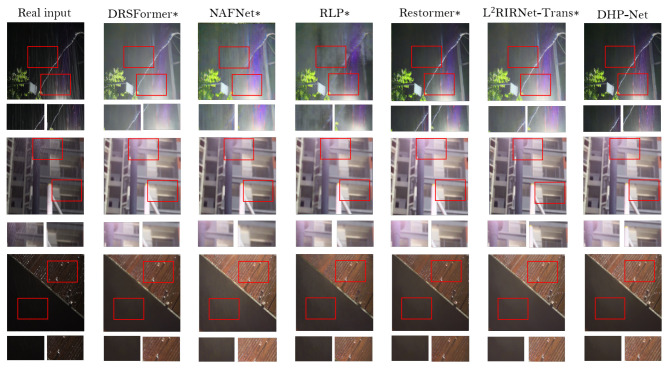
Qualitative comparison on real-world low-light rainy images. Note that no paired ground-truth reference is provided for real-world scenes. Red boxes highlight representative local regions for comparing visibility enhancement, rain suppression, and structural preservation. DHP-Net yields more balanced restoration results with cleaner details and fewer residual artifacts. ∗ Methods marked with an asterisk are retrained on the LLR training set under the same experimental protocol for fair comparison.

For real-world low-light rainy images, the visual comparisons in Figure 4 show that existing methods often suffer from under-enhancement, residual rain artifacts, or excessive smoothing after brightness correction. Deraining-oriented baselines tend to retain dark appearance and miss hidden degradations, whereas enhancement-oriented baselines may amplify noise or create locally over-exposed regions. In contrast, DHP-Net produces more balanced restoration results, improving scene visibility while preserving structural clarity and suppressing rain interference more effectively. These visual improvements agree with the NIQE gains in Table 4, further showing that the proposed method generalizes well beyond synthetic supervision.

**Figure 5:**
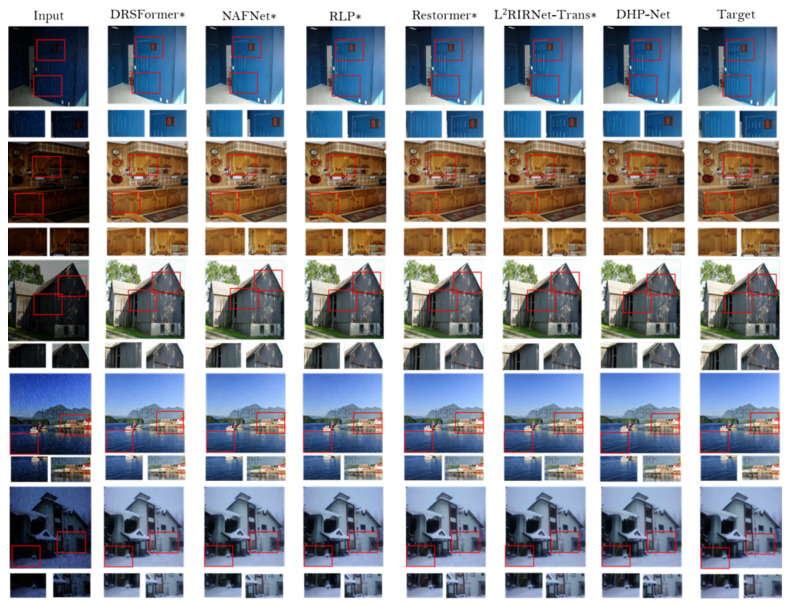
Qualitative comparison on synthetic low-light rainy images. Red boxes highlight key local regions for comparing brightness recovery, rain suppression, and detail preservation. DHP-Net produces more natural illumination correction and clearer structural restoration under coupled degradations. ∗ Methods marked with an asterisk are retrained on the LLR training set under the same experimental protocol for fair comparison.

For synthetic low-light rainy images, Figure 5 shows that DHP-Net restores more faithful brightness and cleaner local structures under coupled degradations. Compared with DRSFormer* and Restormer*, our method leaves fewer residual rain streaks around thin edges and textured regions, while NAFNet* and PairLIE* more often produce over-smoothed details or unstable contrast transitions. In the enlarged regions, DHP-Net better preserves building contours, object boundaries, and scene textures while avoiding the haze-like blur that appears in several competing results. These visual observations are consistent with the quantitative gains reported in Table 2.

### 4.4. Ablation Study

To verify the contribution of each core component in our DHP-Net, we conduct extensive ablation studies on the LLR synthetic test set, with all experiments conducted under the same training and testing settings.

#### 4.4.1. Effectiveness of Key Architecture

We first compare several model variants to validate the overall architecture design. Specifically, V1 denotes the baseline Restormer backbone without any additional modules, V2 adds the channel shuffle mechanism to the baseline, and V3 corresponds to the full DHP-Net with both channel shuffle and the hue prior module. The quantitative results are reported in Table 9.

Compared with V1, introducing channel shuffle improves the PSNR by 0.58 dB and the SSIM by 0.0050, showing that channel reorganization effectively enhances feature interaction and representation quality. Adding the hue prior on top of V2 further improves the PSNR by 0.84 dB and the SSIM by 0.0099, validating the importance of explicit degradation-robust prior guidance for coupled low-light rainy restoration. The full DHP-Net achieves the best performance of 33.14 dB PSNR and 0.9252 SSIM.

#### 4.4.2. Effectiveness of Loss Function

We then validate the effectiveness of our multi-component loss function by comparing different loss combinations. Table 10 summarizes the quantitative results of the ablation study on different loss combinations, verifying the contribution of each loss component. The full loss function consists of L1 loss, VGG perceptual loss, and SWT frequency loss with weights of 1.0, 0.05, and 0.15, respectively. Experimental results show that this combination effectively balances pixel-level fidelity, perceptual quality, and frequency-domain consistency, comprehensively improving restoration performance. Compared with the model trained with only L1 loss, the introduction of VGG loss and SWT loss improves the PSNR by 0.57 dB and SSIM by 0.0149.

#### 4.4.3. Loss Weight Analysis

We further analyze the influence of the balancing weights in the training objective. Table 11 reports the ablation results of different combinations of VGG and frequency-domain loss weights on top of the L1 reconstruction loss. The results show that incorporating either perceptual supervision or frequency-domain supervision alone already improves over pure L1 optimization, while their combination yields the best overall performance. In particular, setting $\lambda_{1}=0.05$ and $\lambda_{2}=0.15$ provides the best trade-off between reconstruction fidelity and perceptual quality, which is why these values are adopted in all experiments.

#### 4.4.4. Effectiveness of the Hue Prior

We further conduct an ablation study on the hue prior module by comparing the model performance with and without this design. Table 12 presents the ablation results of the hue prior module, quantitatively verifying its performance improvement for coupled degradation restoration. The results show that with the hue prior, the model can better preserve the structural and color information of the scene, with PSNR increasing from 32.30 dB to 33.14 dB and SSIM increasing from 0.9153 to 0.9252. This significant performance gain verifies the effectiveness of degradation-robust hue guidance for low-light rainy image restoration.

#### 4.4.5. Effectiveness of Channel Shuffle

Finally, we verify the contribution of the channel shuffle mechanism by comparing models with and without this design. Table 13 presents the corresponding quantitative results. The results show that the channel shuffle mechanism enables the model to utilize cross-channel information more effectively, with PSNR increasing from 32.56 dB to 33.14 dB and SSIM increasing from 0.9153 to 0.9252. This validates that channel shuffle enhances the model’s feature interaction capability and adaptive modulation performance for coupled degradations.

Through the above ablation studies, we comprehensively verify the effectiveness of each core component of DHP-Net, and demonstrate that the synergistic combination of these components achieves optimal restoration performance. The proposed model provides an efficient visual enhancement solution for practical applications including autonomous driving and video surveillance systems.

### 4.5. Component Analysis of CAAPM

To further verify the individual contribution of each design in the proposed Channel-adaptive Attention Perturbation Module, we conduct component-level ablation experiments covering four core variants: branch attention only, perturbation branch only, fixed shuffle strategy, and reduced channel dimension. All models are trained and evaluated under the same protocol with ten quantitative metrics from multiple perspectives to ensure comprehensive and fair comparison.

The detailed results are summarized in Table 14.

Experimental results show that the complete CAAPM design achieves the best performance across all main metrics. Removing the entire module leads to a notable performance drop in both full-reference metrics (PSNR, SSIM, LPIPS) and no-reference perceptual metrics, which confirms that the perturbation-aware feature recalibration mechanism is essential for handling coupled low-light and rain degradations. When only the branch attention or only the perturbation branch is retained, the performance decreases to different degrees, indicating that the two components play complementary roles and their combination enables more effective feature interaction. Replacing adaptive channel shuffle with a fixed shuffle pattern also reduces the overall performance, which demonstrates that input-adaptive routing can adjust feature reorganization according to specific degradation conditions and bring additional gains. In addition, reducing the internal channel dimension of the module causes obvious performance degradation, verifying that sufficient channel capacity is required to model complex entangled feature responses. All metrics show consistent trends, which fully validates the rationality of each component design in CAAPM. From the perspective of computational cost, the complete CAAPM module only introduces 5.21% additional FLOPs to the overall framework, which is a very limited computational overhead compared to the significant performance improvement it brings. This excellent performance–efficiency ratio further demonstrates the practical value of the proposed channel-adaptive perturbation design.

### 4.6. Application to Object Detection

**Figure 6:**
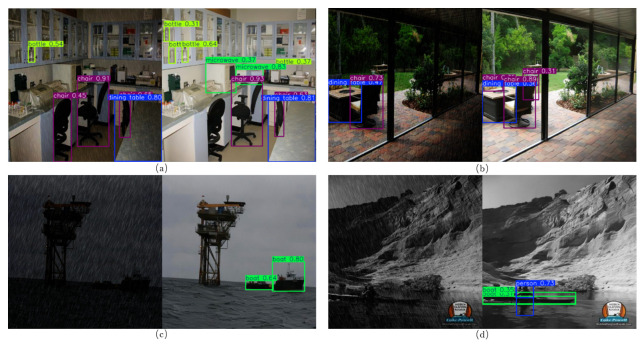
Application to downstream outdoor object detection using YOLOv5s [67]. All examples are selected from real-world low-light rainy street scenes. The labels (**a**–**d**) are shown only for visual illustration and are not described separately. For each example pair, the left image shows the detection result on the degraded low-light rainy input, while the right image shows the detection result after restoration by DHP-Net. Restoration improves object visibility and structural clarity, leading to more complete detections and generally higher confidence scores in adverse scenes.

Beyond low-level restoration quality, DHP-Net can also benefit downstream high-level vision tasks in adverse environments. To provide an application-oriented evaluation, we further visualize object detection results produced by YOLOv5s [67] on degraded low-light rainy images and on the corresponding images restored by DHP-Net. As shown in Figure 6, directly applying the detector to degraded inputs often leads to missed targets, incomplete localization, and low-confidence predictions because rain interference and severe under-exposure suppress discriminative visual cues. After restoration, the scene becomes more observable, object boundaries are clearer, and local contrast is substantially improved, which enables the detector to identify more objects with tighter bounding boxes and more reliable confidence scores. This effect is particularly noticeable for outdoor targets such as pedestrians, vehicles, and traffic signs under poor illumination and low-light rainy scenes, which are frequently missed or weakly detected in the original degraded inputs. These results indicate that DHP-Net is not only effective as a restoration model, but also useful as a pre-processing module for practical perception systems in low-light rainy conditions.

## 5. Conclusions

Low-light rainy scenes widely exist in real-world outdoor scenarios such as autonomous driving, video surveillance, and nighttime traffic monitoring. The coupled degradation of insufficient illumination and rain streak corruption severely degrades the imaging quality of visual perception systems, which directly restricts the reliability of subsequent high-level vision tasks and has become a common bottleneck restricting the all-weather operation of outdoor perception systems. Existing single-task restoration methods and simple cascaded pipelines often fail to handle the mutual interference between brightness enhancement and rain removal, making it difficult to meet practical application requirements.

This paper presents DHP-Net, a single-stage framework for low-light rainy image restoration that combines a degradation-robust hue prior with perturbation-aware Transformer restoration. By injecting multi-scale hue guidance into the hierarchical backbone and recalibrating entangled intermediate responses through channel-adaptive attention perturbation, the proposed method effectively addresses the coupled challenges of brightness degradation and rain corruption.

Different from most existing methods that rely on data-driven degradation features or dual-branch separate modeling, the hue prior design provides stable structural and color guidance independent of training degradation distributions, while the channel-adaptive attention perturbation mechanism resolves conflicting feature responses caused by coupled degradations within a unified single-stage architecture, avoiding the computational redundancy and error accumulation of multi-stage pipelines.

Extensive experiments on both synthetic and real-world low-light rainy images show that DHP-Net consistently outperforms cascaded baselines and retrained state-of-the-art restoration models in both quantitative and qualitative evaluations, demonstrating the effectiveness of combining degradation-robust prior guidance with adaptive feature modulation for coupled-degradation removal.

The ablation studies further verify the independent contribution of each core component, and the downstream object detection application demonstrates that the proposed method can effectively improve the performance of high-level vision tasks in adverse weather. This work not only provides an efficient and robust solution for low-light rainy image restoration, but also offers a new technical reference for the research on coupled mixed-degradation-restoration tasks.

## 6. Discussion and Limitations

Despite the favorable restoration performance achieved by DHP-Net on both synthetic and real-world low-light rainy scenes, the method still has clear limitations and application boundaries. A detailed analysis of failure cases and remaining challenges is provided as follows.

First, the restoration quality degrades noticeably under extremely strong coupled degradation, especially when heavy rain streaks are densely superimposed on severely underexposed dark regions. In such cases, the hue prior also suffers from severe information loss due to extremely low signal-to-noise ratio, and the stable structural cues are weakened; meanwhile, densely overlapping rain streaks will cause the channel-adaptive perturbation module to misjudge local texture features, resulting in residual rain artifacts or blurred edge details. This type of failure is mainly concentrated in nighttime scenes with almost no ambient light and heavy rainfall, which is also a common challenge faced by existing single-image restoration methods.

Second, the current method still has insufficient control over color consistency and local semantic integrity. Since the hue prior mainly provides structural and layout guidance and lacks high-level semantic constraints, the restored results may have slight color deviation in scenes with complex light sources (such as neon lights, street lamps and reflective surfaces), and the texture of small semantic targets (such as far-distance traffic signs and text markers) may be over-smoothed. In addition, for real-world scenes with non-uniform rain distribution, the local restoration strength cannot be dynamically adjusted pixel by pixel, which may cause over-deraining in light-rain regions or residual rain streaks in heavy-rain regions.

Third, from the perspective of practical deployment, the current Transformer-based architecture still has non-negligible computational overhead for edge devices with limited computing power. Although the single-stage end-to-end design avoids the error accumulation of cascaded pipelines, the overall parameter count and FLOPs are still at the same level as mainstream Transformer restoration models. For highly resource-constrained embedded platforms, such as low-power ARM-based edge terminals and embedded visual monitoring sensors, the current inference latency and peak memory consumption still cannot meet the requirements of high-frame-rate real-time processing. In addition, direct model compression operations such as low-bit quantization may also bring unpredictable restoration performance degradation, which brings certain challenges to actual embedded deployment.

Fourth, in terms of generalization scope expansion, we will extend the validation of the proposed framework to more diverse visual restoration benchmarks, including standard low-light enhancement datasets and general mixed-degradation benchmarks, to explore the transferability and universality of the degradation-robust hue prior and channel-adaptive perturbation mechanism across more degradation types and application scenarios.

Fifth, the application scope of the current framework is still limited to the coupled low-light rainy degradation scenario, and its generalization performance under other adverse weather conditions has not been systematically verified. The proposed hue prior and channel-adaptive perturbation mechanism are specially optimized for the coupling characteristics of illumination attenuation and rain streak distortion. However, other common adverse weather such as fog, snow, and dust follow distinct degradation mechanisms: fog causes global contrast attenuation through atmospheric scattering, snow presents multi-scale particle occlusion and local overexposure, and dust introduces uneven color cast and texture blur. The effectiveness of the proposed method under these degradation types has not been validated, which constitutes another clear boundary limitation of this study.

Correspondingly, future work will be carried out around the above limitations. For extreme degradation scenarios, we will explore the introduction of physical degradation modeling and multi-frame temporal consistency constraints to further improve the robustness under severe coupled corruption. For color and semantic accuracy, we will try to integrate semantic priors or diffusion priors from large-scale vision foundation models, so as to provide higher-level guidance for restoration while maintaining lightweight overhead. For deployment requirements, we will carry out research on model compression and efficient architecture design, including knowledge distillation, structured pruning and quantization, to build a more efficient and deployable coupled restoration model. For cross-scenario generalization, we will explore the extensibility of the degradation-robust hue prior and adaptive feature perturbation paradigm to fog, snow, dust and other complex adverse weather restoration tasks to build a more universal multi-degradation adaptive restoration framework. For extreme embedded deployment requirements, we will carry out in-depth research on lightweight architecture design and end-side deployment optimization, including structured pruning, INT8 quantization and operator-level hardware adaptation, to realize real-time inference on low-power edge chips.

## Figures and Tables

**Table 1:** Channel-wise stability comparison between degraded and clean images (800-sample test set).

Channel	SSIM	Mutual Information	Entropy Stability
R	0.4333±0.1442	2.2511±0.5494	−1.1838±0.7436
G	0.4207±0.1469	2.2169±0.5673	−1.2436±0.7700
B	0.3924±0.1482	2.0449±0.6033	−1.4441±0.8500
H	0.7714±0.1369	1.7994±0.6763	−0.3207±0.4228
S	0.6166±0.1421	1.6273±0.5938	−0.5071±0.6234
V	0.4597±0.1410	2.3374±0.5051	−0.9971±0.6928

Note: Values are presented as mean ± standard deviation. One-sided paired *t*-tests with Holm family-wise correction confirm that the hue (H) channel has significantly higher SSIM and entropy stability than all other channels (all adjusted p<0.001).

**Table 2:** Quantitative comparison results.

Methods	PSNR (dB)	SSIM	Param (M)	Running Time (ms)
**Cascaded Methods **				
Zero-DCE ⇒ RCDNet	17.58	0.6634	3.5	235.33
SCI ⇒ MIRNet	20.71	0.7186	32.3	198.50
UHDNet ⇒ Restormer	19.91	0.6928	59.8	206.64
PairLIE ⇒ DRSFormer	18.29	0.6495	34.0	555.38
MIRNet ⇒ SCI	19.32	0.6931	32.3	198.50
RCDNet ⇒ Zero-DCE	12.21	0.6175	3.5	235.33
Restormer ⇒ UHDNet	19.57	0.6878	59.8	206.64
DRSFormer ⇒ PairLIE	19.19	0.6868	34.0	555.38
**Retrained Methods**				
Zero-DCE*	21.75	0.7189	0.5	57.81
RCDNet*	25.04	0.8031	3.0	177.52
MIRNet*	29.31	0.8872	31.8	193.53
Restormer*	31.19	0.9174	26.1	190.53
NAFNet*	29.17	0.8987	17.1	32.67
PyDiff*	29.06	0.8753	54.5	373.56
UHDNet*	24.91	0.7914	33.6	16.11
PairLIE*	19.33	0.6948	0.3	2.42
RLP*	29.45	0.8878	5.3	101.72
DRSFormer*	31.58	0.9182	33.7	552.96
$L^{2}$RIRNet-CNN*	30.53	0.9030	73.9	58.38
$L^{2}$RIRNet-Trans*	32.53	0.9231	37.5	229.01
**DHP-Net (ours) **	**33.14**	**0.9252**	35.6	207.46

* Methods marked with an asterisk are retrained on the LLR training set under the same experimental protocol for fair comparison.

**Table 3:** Full multi-metric comparison between DHP-Net and state-of-the-art mixed-degradation-restoration methods on synthetic LLR test set.

Metric	AdaIR	PromptIR	DHP-Net (Ours)
PSNR ↑	30.8197	30.8413	33.1400
SSIM ↑	0.9052	0.9215	0.9252
LPIPS ↓	0.0550	0.0705	0.0415
NIQE ↓	3.6221	3.6036	3.6400
BRISQUE ↓	21.1094	21.5116	22.3000
MUSIQ ↑	66.0818	66.0402	66.6800
CLIPIQA ↑	0.4616	0.4632	0.4878
PIQE ↓	37.3881	38.1871	39.4200
LOE ↓	0.1384	0.0195	0.1260
Entropy ↑	7.3258	7.3826	7.3300

Note: All models are fully retrained on LLR training set with identical training hyperparameters. AdaIR takes results at Epoch 90; PromptIR takes results at Epoch 100. ↑ = higher better, ↓ = lower better.

**Table 4:** Quantitative evaluation on real-world LLR images.

Methods	NIQE ↓	Methods	NIQE ↓
Input	16.93	DRSFormer*	14.82
RCDNet*	15.97	RLP*	14.61
MIRNet*	14.31	PairLIE*	17.34
Restormer*	15.55	$L^{2}$RIRNet-Trans*	14.17
NAFNet*	14.65	**DHP-Net (ours)**	**13.42**

* Methods marked with an asterisk are retrained on the LLR training set under the same experimental protocol for fair comparison. The downward arrow ↓ indicates that lower values correspond to better image quality.

**Table 5:** Multi-dimensional quantitative comparison on the LLR synthetic test set.

Metric	DHP-Net (Ours)	$L^{2}$RIRNet-Trans*	DRSFormer*	Restormer*
*Full-reference metrics *				
PSNR (dB) ↑	33.14	32.53	31.58	31.19
SSIM ↑	0.9252	0.9231	0.9182	0.9174
LPIPS ↓	0.0415	0.0463	0.0813	0.0658
*No-reference metrics*				
NIQE ↓	3.64	3.24	3.61	3.66
BRISQUE ↓	22.30	17.61	19.57	22.40
PIQE ↓	39.42	31.63	34.13	39.65
LOE ↓	0.1260	0.0384	0.0408	0.0503
Information Entropy ↑	7.33	7.36	7.39	7.35
MUSIQ ↑	66.68	65.67	65.47	66.70
CLIP-IQA ↑	0.4878	0.4906	0.4469	0.4887

Note: ↑ = higher better; ↓ = lower better. * denotes methods retrained on the LLR training set.

**Table 6:** Full quantitative results on extended test sets for generalization verification.

Metric	Original LLR Test Set	+ Rain100L Extended	+ Rain100H Extended
PSNR (dB) ↑	33.14	29.43	31.49
SSIM ↑	0.9252	0.8901	0.9163
LPIPS ↓	0.0415	0.0679	0.0480
NIQE ↓	3.64	3.63	3.63
BRISQUE ↓	22.30	21.03	22.34
MUSIQ ↑	66.68	65.15	66.40
CLIP-IQA ↑	0.4878	0.4343	0.5046
PIQE ↓	39.42	38.84	41.66
LOE ↓	0.1260	0.1286	0.1307
Information Entropy ↑	7.33	7.34	7.34

The upward arrow ↑ indicates higher values correspond to better performance; the downward arrow ↓ indicates lower values correspond to better performance.

**Table 7:** Computational efficiency of DHP-Net at different input resolutions.

Input Resolution	FLOPs (G)	FPS	Latency (ms)	Peak Memory (MB)
256 × 256	168.81	15.73	63.56	743.06
512 × 512	675.22	3.47	288.20	2573.10

**Table 8:** Computational complexity breakdown of the CAAPM module (256 × 256 input).

Component	Parameters (M)	FLOPs (G)
Full DHP-Net	31.72	168.81
Total CAAPM	4.98	8.79
Encoder stage 1	–	2.94
Encoder stage 2	–	2.68
Encoder stage 3	–	2.55
Latent bottleneck	–	0.62
CAAPM proportion	15.70%	5.21%

**Table 9:** Ablation study on key architecture.

Variant	Channel Shuffle	Hue Prior	PSNR (dB)	SSIM
V1	×	×	31.72	0.9103
V2	✓	×	32.30	0.9153
**V3 (Ours) **	✓	✓	**33.14**	**0.9252**

**Table 10:** Ablation study on loss function.

Loss Combination	PSNR (dB)	SSIM
L1	32.57	0.9103
L1 + 0.05VGG	32.85	0.9153
**L1 + 0.05VGG + 0.15SWT (Ours) **	**33.14**	**0.9252**

**Table 11:** Ablation results of loss weight combinations.

Group	L1 Loss Weight	VGG Loss Weight	SWT Loss Weight	PSNR (dB)	SSIM
1	1.0	0.00	0.00	32.57	0.9103
2	1.0	0.05	0.00	32.89	0.9187
3	1.0	0.00	0.15	32.76	0.9165
4	1.0	0.05	0.10	33.01	0.9228
5	1.0	0.05	0.15	33.14	0.9252
6	1.0	0.10	0.15	33.09	0.9239

Note: Group 1 uses only pixel-level L1 loss as the baseline. Group 2 and Group 3 individually introduce VGG perceptual loss and SWT frequency-domain loss to verify their independent contributions. Group 4 and Group 5 explore different weight combinations of the two auxiliary losses, where Group 5 is the final optimal configuration adopted in this paper. Group 6 further increases the perceptual loss weight to verify the performance degradation caused by over-strong perceptual supervision.

**Table 12:** Ablation study on the hue prior.

Model Configuration	PSNR (dB)	SSIM
w/o Hue Prior	32.30	0.9153
**w/ Hue Prior (Ours)**	**33.14**	**0.9252**

**Table 13:** Ablation study on channel shuffle.

Model Configuration	PSNR (dB)	SSIM
w/o Channel Shuffle	32.56	0.9153
**w/ Channel Shuffle (Ours) **	**33.14**	**0.9252**

**Table 14:** Component ablation study of the CAAPM module.

Model Configuration	PSNR ↑	SSIM ↑	LPIPS ↓	NIQE ↓	BRISQUE ↓
Full CAAPM (complete design)	33.14	0.9252	0.0415	3.64	22.30
w/o entire CAAPM module	30.91	0.9057	0.0488	3.52	22.18
w/o perturbation branch (branch attention only)	29.59	0.8985	0.0586	3.60	21.75
w/o branch attention (perturbation branch only)	29.59	0.8976	0.0600	3.65	20.87
w/ fixed shuffle (replacing adaptive routing)	29.53	0.8949	0.0633	3.62	21.53
w/ reduced channel dimension	28.93	0.8834	0.0791	3.70	22.18
**Model Configuration **	**MUSIQ** ↑	**CLIP-IQA** ↑	**PIQE** ↓	**LOE** ↓	**Entropy** ↑
Full CAAPM (complete design)	66.68	0.4878	39.42	0.1260	7.33
w/o entire CAAPM module	66.18	0.4645	40.34	0.1300	7.36
w/o perturbation branch (branch attention only)	65.76	0.4733	39.94	0.1306	7.36
w/o branch attention (perturbation branch only)	65.90	0.4523	39.15	0.1493	7.35
w/ fixed shuffle (replacing adaptive routing)	65.60	0.4828	38.66	0.1292	7.35
w/ reduced channel dimension	63.74	0.4294	38.12	0.1532	7.35

The upward arrow ↑ indicates higher values correspond to better performance; the downward arrow ↓ indicates lower values correspond to better performance.

## Data Availability

The LLR dataset used in this study was introduced by $L^{2}$RIRNet [20]. Further data and implementation details are available from the corresponding author upon reasonable request.

## Abbreviations

The following abbreviations are used in this manuscript:

Adam	Adaptive Moment Estimation
BIQA	Blind Image Quality Assessment
BRISQUE	Blind/Referenceless Image Spatial Quality Evaluator
CAAPM	Channel-adaptive Attention Perturbation Module
CLIP-IQA	CLIP-based Image Quality Assessment
CNN	Convolutional Neural Network
DHP-Net	Degradation-Robust Hue Prior Network
FFN	Feed-Forward Network
FLOPs	Floating-Point Operations
FPS	Frames Per Second
HFE	Hierarchical Feature Extraction
HSV	Hue, Saturation, Value
LLR	Low-Light Rain
LOE	Lightness Order Error
LPIPS	Learned Perceptual Image Patch Similarity
MI	Mutual Information
MUSIQ	Multi-Scale Image Quality
NIQE	Natural Image Quality Evaluator
PIQE	Perception-based Image Quality Evaluator
PSNR	Peak Signal-to-Noise Ratio
RGB	Red, Green, Blue
SD	Standard Deviation
SSIM	Structural Similarity Index Measure
SWT	Stationary Wavelet Transform
VGG	Visual Geometry Group
